# New York State Veterinary Medical Association

**Published:** 1892-02

**Authors:** N. P. Hinkley

**Affiliations:** Secretary


*New York State Veterinary Medical Society.*—The second annual meeting of the New York State Veterinary Medical Society was held in the assembly room of the Vanderbilt House, Syracuse, N. Y., on Tuesday, January 12th, 1892. The meeting was called to order at 10 A. M. by the President, Dr. C. D. Morris. The roll-call by the secretary showed a very fair percentage of the members present, several members came in later in the day, also members of the profession from different parts of the State who were not members of the Society.

PRESIDENT MORRIS' ADDRESS.

President Morris in his annual address said that he was especially gratified by the energy shown by the members. The Society has been in existance but two years. In that short length of time it has become known throughout the profession in the State, and this acquaintance and knowledge of the society, whether direct or indirect has left the impression with the practitioner that the Society is marching with aggressive pace upon the ignorant and superstitious methods as annunciated by the forefathers and the empirics of the day; this may seem unnecessary, but if we progress we must reform old methods, if our science advances we must put into practice the product of our experience, and into words, the evolution of conscious thought "that men may rise on stepping stones of their dead relics to higher things." The constant endeavor of the officials connected with this society has been to place the profession upon the high standard which it justly merits, and the efforts that have been put forth in the way of reform, to some, may seem arbitrary and unwarranted, at this stage and history of the profession. But if the profession ever expects to enjoy the immunity, dignity and respect that our sister professions are enjoying we must commence at once ourselves, and that start must be on the lines other those which are purely mercenary. It is too often the case that we foot the sum total of a month's work on the number of dollars made, more than on the real service rendered.

It has been my privilege, during the past year, to visit some of the veterinarians in the State, some of whom were members of this Society, and others not members of any. And to my surprise, I found that many of these gentlemen were not taking a veterinary journal. Some were subscribers to only one. Gentlemen, in a spirit that is neither criticism nor reproach, this is the dearest economy that can possibly be practiced. The times demand that we stand abreast of them. No physician can practice with success who is not a reader of the current literature of his profession, and if we expect to be successful practitioners, we must study the latest evolution of thought and practice. The springing up of new schools in the country is not so much on account of a demand for a greater number of institutions teaching the art, as it is to offer facilities for an easier method of obtaining a degree. The old-established colleges and universities of our country are quite capable of taking care of the demand at the present time; they are in a position to offer inducements which are superior to any new school; their experience, reputation and faculty are a sufficient guarantee of their fitness and ability. It seems to me, with these facts in view, that the tendency of this sort of education is to lower the character of the profession, and to place a class of practitioners before the public who will be holding degrees and passing as qualified men, yet morally who are but very little above, if not on a par, with the empiric of the day, seems to me to demand arbitrary restrictions upon the profession to the extent requiring a thorough preparation, to the end not merely of obtaining a degree, but that the student is in possession of a thorough college training. The bill which this Society has placed before the present legislation possesses certain merits. It presumes that the student coming from an institution requiring a four years' course of six months each of continuous study is thoroughly qualified so far as lies within the power of any institution to qualify. It was not the intent of the framers of the act to strike at the heart of any of the established institutions. It was a matter of serious reflection, knowing that within the border of our State there exists one of the best schools on the American continent, whose Alumni many of whom hold positions of professional honor and trust in the Federal Government, whose aim has been to emulate those principles and bring to the surface the product of scientific research, the best fruits and experience the profession affords to day, their able corps of instructors, and particularly her Dean, whose idea has been to reach the highest standard attainable, and yet to exempt the graduates of this institution would be to class legislation, and to my mind would not only prove disastrous to the bill, but to the interest of the same. I feel justified in the position I take regarding the colleges.

While I would in no way dictate matters which to them are of an individual nature, and assume in any way to know what to them are vital interests as a corporation in which I have no part, yet I do presume that should a demand arise in the profession from this State, asking that the institutions teaching the art extend their curriculum to four years of six months each with a more elaborate exhaustion of the science, it would not only be a science of profit to the colleges, but it would establish the character of the profession upon a profound basis. Such a step would be the initiative of a broad principle, resulting in a higher education of the profession. The public is demanding on every side that the status of art and science, as is taught in our institutions of learning, be made higher.

During the last decade the higher institutions of learning have been requiring a more rigid matriculation, and establishing a thorough curriculum.

The members were greatly pleased with the able manner in which President Morris pointed out the matters of vital importance to every lover of the profession regarding legislation, and loudly applauded him for the same. The reading of the minutes of the last meeting was dispensed with for want of time.

President Morris then asked for all present, who were not members of the Society, and wished to become members, to make application to the Censors. Fifteen minutes' recess was given for the same. The following applications were filed with the Censors: R. A. McLean, D. V. S., Brooklyn; W. J. Wadsworth, V. S.; D. H. Rowe, V. S.; H. W. Skerritt, V. S.; F. Marrow, V. S.; V. L. James, V. S.; A. H Ide, V. S.

President Morris called for the report of Committees on Arrangements. Dr. Hinkley answered that no report was to be made. The President called for the report of Committee on Publication. Dr. Hinkley. Secretary, replied that he had included the report in his address, which would be read later. President Morris made the following short report of Committee on Legislation, "being a member of that committee." He said: There has been a bill drafted, printed and copies of it sent to each member of this Society, and also to some members of the profession, whom we know to be qualified men. He then explained the texture of the bill, and said it would be presented to the Legislature at this session. He hoped it would be introduced on evening of January 12, 1892. He said the bill had been thoroughly discussed at the meeting in New York, August 12, 1891, by all members present, including Professors Law and Liautard. He also said there was no need of a man to practice as a Farrier, if he wanted to be a veterinary surgeon, as there was every opportunity offered him to qualify by graduating from a veterinary college. *Dr. H. Sutterby:* I move report be received and placed on file. Motion seconded by Dr. John A. Bell. Voted on, carried *Dr. H. Sutterby:* I move we pass resolution of thanks to Dr. Morris. *Dr R. A. McLean:* I amend to include Legislative Committee. Seconded, and carried unanimously. *Dr. Morris:* Committee on By-Laws please report. *Dr. Hinkley:* Would advise revision of by-laws to be reported at next meeting. Would also advise that a Committee on By-Laws be appointed, all members of this Committee be residents of same county, or portion of the State, that they may be able to meet and act at any time without being obliged to travel a long distance. *Dr. R. A. McLean:* I think an applicant for membership should be made acquainted with by-laws before paying fees and dues, as called for in Section 8. President Morris then called upon the Secretary for his report, which was read, as follows:

### REPORT OF THE SECRETARY.

The past year of 1891, I am pleased to tell you, has been one of prosperity, success and encouragement to every qualified Veterinary Surgeon in the State of New York, and especially to the members of this Society. We meet again to-day with new life, new blood, and strong reinforcements to our ranks of the best and most active of workers in the veterinary profession. Gentlemen who in the past have been somewhat averse to becoming members or co-workers in any society pertaining to veterinary science, because through some hook or crook the non-qualified or quack would creep in, one by one, until they were in the majority, and the result was always the same. Oil and water, acid and alkali, will not mix without considerable effervesence, and the same can be said of the qualified and the quack. And usually the society has become extinct. When this Society was first organized, only two years ago, in the City of Bochester, a mere handful of men, twenty-seven in all, from all parts of the State, were present. We were few in number, but earnest in our cause, and willing to work and wait for our aims and ambitious desires for the better protection of the qualified Veterinary Surgeons of the Empire State, and have a line of demarcation between the graduated Surgeon and the charlatan. To-day we have fifty-seven members in good standing, who are active and persevering in this good work, and numerous more who are knocking at our door for entrance, who are sincere in their workings for the profession. Surely you will agree with me when I say that this is a sure sign of prosperity, and of a healthy and successful Society. Should the present ratio of new members continue during the next five years, you will have a membership roll of nearly 500 members. Why should we not feel proud of this, the Empire State of the Union? Will our brothers in the profession not compete with any in the United States, either in ability or professional learning? We have two colleges and a chair in veterinary surgery that will compete with any, either American or foreign, when we have the hearty co-operation of such hard and earnest workers in the science as Professors Law and Liautard and numerous others who are growing up in the profession with all of the advantages of society, education and proper training, with the young blood of youth coursing throngh their veins, willing to work and labor for the good cause. Why should we not feel encouraged when we stop to think that upon the shoulders of these very same men will be placed the great responsility of moulding and guiding the future of our veterinary structure in America? We should feel doubly pleased to see that our Government has already in national sanitary work commenced to feel the want of better veterinary inspection of our meats and live-stock, to not only place us on an equal footing with our sister countries, but, American like, to be at the head. We should aim to be placed at the head of all nations as the first sanitary protection of food and food products. Sanitation, in its broad and important meaning, should receive careful and thoughtful consideration on our part, that the public sentiment may be aroused and give us our proper place as sanitarians. In veterinary jurisprudence, let us hope that the time is not far distant when our now almost useless and inferior laws may be cast aside, and more broad and fair statutes take their place, and remove from our respected profession the very unjust criticism that makes us the laughing stock of some of our patrons. The rapid and advanced strides that our country has taken during the last four years in the breeding of fine and blooded stock should stimulate us to making ourselves close students to breeding in all its many conditions. Veterinary education, in this we should all agree, and make the effort of our life to reach the standard of our schools, and to watch their welfare and prosperity with a zealous eye, and to lend our aid to widen and improve their standard of education. Our veterinary journals, I must admit, are few in number, but we feel proud of what we have got as being edited and conducted and published by a few of the most learned, scientific and self-sacrificing men that the veterinary profession can boast of. And I for one would feel like going without one of my daily meals, rather than to be deprived of the valuable information and opinions expressed in an American veterinary journal.

Gentlemen, in my official capacity as Secretary to your Society during the past two years, I have had every opportunity to become acquainted with many veterinarians in New York State, if not in person, through correspondence, and it gives me great pleasure to state that, although many in number and scattered in location, all seem to feel the necessity of banding together for our mutual protection and the protection of the suffering public. I find that in our crude and hurried construction of our by-laws, that there is considerable room for improvement, and would suggest that the same be carefully considered at this meeting and revised as the committee may decide. The same may be said of the Code of Ethics. I would also suggest that our President, in naming his several committees, to have the members of a committee selected from that location near each other that they may meet oftener without having to travel from home, as heretofore committees have been on paper only, as one man was at the eastern part of the State and another at the extreme western part, making it almost impossible for them to meet only by correspondence. This, in my opinion, is detrimental to the interests of the Society, and I trust the President will respectfully ask all such committees to accept the office and to fulfill every obligation, remembering we must all do our share in this great work ahead of us to warrant us success in any part.

As for our financial condition, you are all aware, by the several letters sent out, that I have endeavored to keep all dues and moneys among the Society kept paid, as we are youug in age, aud in starting all societies there is considerable expense attached, which, if all members respond promptly, makes it more easy and pleasant for all concerned. I have endeavored, by printed matter and by personal correspondence, to keep a lively interest with all members, and to try and get all to become personally interested with the workings of the Society. I have compiled for your use a list of nearly all the graduates in Veterinary Medicine and Surgery in New York State, numbering about 300, and I hope my successor will continue to add from year to year new names, to have on hand a reliable directory.

Following in the footsteps of our parent organization, the U. S. Veterinary Medical Association, no members of the profession can become one of our members without first filling out a blank application, and he must be vouched for by two of our members, and show his qualifications, and filing out and signing a blank certificate showing his willingness to obey and abide by our by-laws and constitution. I also suggest that we have the much-needed Comitia Minora appointed by our President, and to further the interest of our Society, that the President appoint County Secretaries in each county; when we have a Secretary in a county, he to report at our annual meeting the condition of the veterinary profession in county, also any special disease or outbreak or anything of importance to our profession, thereby assuriug us of a complete report of all that transpires in the several counties. During the last year I have received and answered nearly 300 letters. I have sent out 450 invitations, 450 programmes, 400 circulars, nearly 100 postal cards, 18 telegrams, and have written nearly 250 personal letters to try and interest all qualified members of our profession in our work, and have sent out 500 copies of our proposed act to regulate veterinary practice in this State. I attended the meeting of the United States Veterinary Medical Association, and felt richly rewarded, for there I met the first organization of veterinary profession in America, and the numerous papers that were read and the discussions which followed brought forth many new ideas, and showed the good work of this grand Society. I would most heartily and sincerely ask that all our members make application for membership in the U. S. Veterinary Medical Association, as we should all feel proud of its good work and lend it our personal aid and encouragement. Before closing my report, I would like to add that the office I now occupy is, and will continue to be, one of great expenditure of time and labor, and as I have served you faithfully and earnestly in this capacity for two years, no inducement you might bring forth would prevail upon me to accept a re-election. I therefore respectfully ask that you will choose from among your members one who will become as much interested and wrapped up in the duties of the office as I have, and finish and continue to make more complete the work I have commenced. I would therefore recommend that some compensation be allowed my successor. I now thank you all very kindly for your personal aid in my labors, and especially to the officers of this Society do I feel grateful for their generous support. I have here my vouchers for expenses during the past year, and respectfully submit my report. Thanking you for your kind attention, I remain fraternally yours, N. P. HINKLEY, D. V. S., *Secretary.*

*Dr. H. Sutterby:* I move the Secretary's report be accepted. Motion seconded by Dr. Jno. Wende. Voted on. Carried unanimously. President Morris then made a few remarks about the amount of work attached to the Secretary's office, and that he is justly entitled to remuneration. *Dr. Hinkley:* I do not want any pay for services already rendered, but would suggest that my successor receive remuneration, that the work may be properly done. *Dr. Morris:* I make a motion that the Secretary be empowered to appoint an assistant at a salary of one hundred dollars a year. *Dr. H. Sutterby:* I amend that motion to read that the Secretary appoint W. B. Drake as his assistant at a salary of one hundred dollars a year. Motion, as amended, seconded and carried unanimously. *Dr. Morris:* What is the opinion of the members about having a Secretary appointed in each county, and also a Comitia Minora? *Dr. H. Sutterby:* I think it unnecessary to have a Comitia Minora; I think their decisions would be overridden by the members of the Society at the meetings. *Dr. Jno. Wende:* I think a Comitia Minora all right; it would cut short the labors of committees. *Dr. Morris:* If we invest them with the power the same as the Board of Censors the two would work together harmoniously. *Dr. Morris:* Have them work together and check all applications for membership that are not all right. *Dr. McLean:* The Comitia Minora in the United States Veterinary Medical Association works very faithfully and freely, going any distance at all times to attend to their callings. *Dr. H. Sutterby:* I think the attempt of any intruder to enter the Society will be promptly met and taken care of as in former meetings by the Board of Censors; I believe in making it their duty. Do not have too many committees, it is expensive. *Dr. Hinkley:* I believe it would be a benefit to this Society to have a Comitia Minora. Let every one speak his opinion and all take an interest. *Dr. Morris:* In some respects Dr. Sutterby's idea is all right. But, as Dr. McLean says, it is of vital importance that we have a Comitia Minora to report at the meetings of this Society matters which do not affect the public and should not be published broadcast. What have the members decided upon regarding appointing a Secretary in each county. *D. H. Sutterby:* I think it a grand idea; it will bring before the proper authorities the condition of the live stock in their several counties, and also help the Veterinarian in his location in a sanitary point and bring him before the public. *Dr. Carnrite:* I agree with Dr. Sutterby, something must be done to protect the public from diseased meat and milk. Dr. Carnrite then cited cases of tubercolosis in his vicinity where milk was being sold from the cows when he discovered them. Dr. Sutterby also cited cases where he had been called on to inspect cows in his vicinity and condemned them, and so reported it to the Board of Health. *Dr. Skillett:* I am milk inspector in my vicinity and condemn all cows suffering from tubercolosis. Dr. McLean cited several instances where he had condemned cattle when on the Board of Health and hoped to see a Veterinarian appointed on every State Board of Health. *Dr. Hinkley:* We ought to have Veterinary Surgeons on every Board of Health in our cities. Dr. Morris cited cases where he condemned and had killed 5 cows out of a herd of 45. The owner was selling milk to the public until reported to the Board of Health by Dr. Morris He also cited a case where a farmer killed hogs suffering from tubercolosis, fed the carcases to other hogs which were killed and the meat sold to the public. Dr. McLean cited a case in Orange county where a milkman whose whole herd were suffering from tubercolosis. When told of it he would not stop selling the milk until his customers were notified and left him, when he killed the whole herd. *Dr. Hinkley:* If we had the legal power to stop this and the medical men would co-operate with us we could save a great amount of sickness and suffering in the human race.

The meeting adjourned until 2 P. M. to allow members to get dinner.

Afternoon session 2 P. M.

*President Morris:* The Secretary will please read his financial report for the past year. Read and accepted. *Dr. H. Sutterby:* I move that the Secrerary notify all delinquent members to pay up at once or their names will be dropped from the roll and they will be ineligible to membership until all arrears are paid. *Dr. McLean:* I second that motion. Voted on and carried unanimously. *Dr. Jno. A. Bell:* I move that President Morris name the men to be known as county secretaries. *Dr. W. L. Baker:* I second the motion. Carried. *President Morris:* We will have fifteen minutes recess to allow all who desire to make application for membership, and the payment of fees and dues.

*President Morris:* We will proceed to the election of officers for the ensuing year. Nomination for President in order.

*Dr. Jno. Wende:* I nominate Dr. R. A. McLean for President. Seconded by Dr. Jno. A. Bell. *Dr. MeLean:* I positively refuse to act. If you desire to go east I will nominate Dr. R. S. Huidekoper. Seconded by Dr. W. L. Baker. *Dr. McLean:* I make motion that the Secretary cast the ballot for Dr. R. S. Huidekoper as President of this Society for the ensuing year. *Dr. H. Sutterby:* Second the motion. Carried. The Secretary cast the ballot. *Dr. Hinkley:* I nominate Dr. H. Sutterby for Vice-President. *Dr. Sutterby:* I nominate Dr. Jno. Wende for Vice-President. No other nominations being made 23 votes were cast, Dr. Sutterby received 11, and Dr. Wende 12. Dr. Jno Wende was declared elected. Dr. Wende was called to the chair. *Dr. H. Sutterby:* I nominate Dr. Hinkley for Secretary for the ensuing year. *Dr. Morris:* I second the nomination. Unanimously elected by verbal vote. *Dr. Sutterby:* I move the Treasurer cast the ballot electing Dr. Hinkley Secretary for the ensuing year. Ballot cast. *Dr. Morris:* I nominate Dr. H. Sutterby for Treasurer. *Dr. Jno. A. Bell:* I second the nomination. *Dr. McLean:* I move the Secretary cast the ballot electing Dr. H. Sutterby Treasurer of this Society for the ensuing year. Ballot cast. *Dr. McLean:* I make a motion that the present President appoint a committee of five to revise the by-laws. The following were appointed :

Drs. Jno. A. Bell, R. R. Bell, R. A. McLean, H. Sutterby and L. E. Willyoung. *Dr. McLean:* I make a motion that the following members be appointed as Board of Censors : Dr. Jno. A. Bell, Chairman ; Dr. James Chase, Dr. John Wende, Dr. V. R. James, Dr. O. B. French. Motion carried and the Secretary cast the ballot electing the above named gentlemen as Board of Censors for the ensuing year. This closed the election of officers. The committees will be appointed by President-elect R. S. Huidekoper.

Dr. Morris then read a copy of the act presented to the Legislature and explained its merits and the necessity of keeping up the standard of the profession and placing it on an equal standing with that of England and with the medical men of this country. The wording in different sections of the act were changed somewhat, not, however, affecting the good merits of the bill. After making the changes it was moved to leave the bill as it read. Voted on and carried unanimously. *Dr. McLean:* I make motion that same committee on legislation be empowered to act as special committee to urge the passage of the bill. Motion seconded and carried. The following comprise the committee : Drs. C. D. Morris, Geo. H. Berns, N. P. Hinkley, R. R. Bell, Prof. Liantard and Prof. Law. Adjourned for supper.

The evening session was called to order at 7.45 P. M. President Morris called on Dr. James Carnite to read a paper on Indigestion. Dr. Carnite presented the subject from a practical standpoint and brought out many valuable facts. He said that after many trials and the loss of numerous patients he was forced to abandon the old orthodox treatment, and set about to find a more efficient method, and considered that he had made a great improvement. He denounced the purgative ball, linseed oil, turpentine and alkaline treatment, with injection pump included, for many reasons. Concluding the disease was due to an acid condition of the stomach, causing a fermentation and generation of gases, he had set about to find a quick and efficient remedy, just to allay pain, and had found nothing to equal chloroform. To stop fermentation and expel gases he had found nothing that equals dilute carbolic acid. Usually he had found that cases yield to one dose of the above remedies. A discussion on Dr. Carnrite's paper followed, in which all members took anactive part.

President Morris called on Dr. James M. Chase to read a paper upon paralysis. *Vide fol.* 85. All members were deeply interested in discussing Dr. Chase's paper.

Dr. Morris read a synopsis of his experience and the gratifying results obtained from the use of pyoktanine in his practice.

*Dr. McLean:* Where will the next meeting be held. *Dr. Morris:* At Syracuse, between July 5 and 15th, 1892. Dr. Hinkley made a few well chosen remarks, highly complimenting Dr. C. D. Morris on his success in starting the New York State Veterinary Medical Society, and the ability he had displayed in the manner in which he had filled the office of President, and I move that Dr. Morris be given a vote of thanks for his able services. Motion seconded by Dr. H. Sutterby and carried unanimously. *Dr. Summerfeldt:* I think we ought to bring to task Drs. Wm. Somerville, Robert M. Somerville, and Wm. Somerville, Jr., regarding a breach of the Code of Ethics, in preparing, advertising and selling proprietary medicines. I have here three circulars that have been printed since Nov. 28, 1891, at which time Dr. Wm. Somerville was admitted to membership in this Society. *Dr. McLean:* I move that the Secretary be instructed to notify the Drs. Somerville to appear before the Board of Censors at the next meeting for a hearing and explain their position in this matter. Motion seconded. Voted on and carried.

After a few remarks by the President, Secretary and other members of the Society a motion was made and seconded to adjourn until the next semi-annual meeting, subject to the call of the Secretary. There was more interest taken in matters brought before this meeting than at any previous meeting held by this Soeiety. Legislative matters received especial attention by all members present.

N. P. HINKLEY, D. V. S., *Secretary,*

